# Intraoperative Pulmonary Embolism Diagnosed by Rescue Transesophageal Echocardiography in a Morbidly Obese Patient Undergoing Orthopedic Surgery following Motor Vehicle Crash

**DOI:** 10.1155/2019/2429194

**Published:** 2019-05-26

**Authors:** Patrick H. Lam, Adam J. Milam, Eric J. Ley, Roya Yumul, Omar Durra

**Affiliations:** ^1^Department of Anesthesiology, Cedars-Sinai Medical Center, Los Angeles, CA, USA; ^2^Department of Trauma Surgery, Cedars-Sinai Medical Center, Los Angeles, CA, USA

## Abstract

A case of intraoperative pulmonary embolism diagnosed by rescue transesophageal echocardiography in a morbidly obese patient undergoing orthopedic surgery following motor vehicle crash, who developed acute and persistent tachycardia, hypotension, and reduction of end-tidal CO2 during general and regional anesthesia, is described.

## 1. Introduction

Surgery and major trauma increase the risk of pulmonary embolism (PE), with a higher risk in those immobile, obese, and smoking. The diagnosis of PE can be challenging, especially in patients receiving general anesthesia. The most common findings are tachycardia, hypotension, hypoxemia, and abrupt reduction of end-tidal CO2 (EtCO2), but PE is considered a diagnosis of exclusion, and current guidelines for intraoperative diagnosis are not well-defined [1–4]. We describe our management of PE diagnosed intraoperatively by rescue transesophageal echocardiography (TEE) in a morbidly obese patient undergoing orthopedic surgery following a motor vehicle crash.

## 2. Case Report

A 28-year-old female with a history of morbid obesity (BMI 50), type 2 diabetes, and a recent unrelated left knee injury and ankle sprain that was managed nonoperatively presented after being struck by an automobile traveling approximately 25 mph and an initial loss of consciousness. The initial vital signs were HR 116, BP 103/85, RR 24, and O2 saturation 100% on room air. The initial chest X-ray (CXR) was within normal limits. The patient was noted to have bilateral nasal bone fractures and a right L2 transverse process fracture which were managed nonoperatively. Other injuries included fractures of the left clavicle, left fibular neck, and left ankle; she was scheduled for open reduction and internal fixation (ORIF) of the left clavicle and left ankle, under regional anesthesia (with interscalene/superficial cervical plexus and popliteal blocks), and general anesthesia to secure her airway due to her BMI and trauma history (risk of aspiration). Induction (with midazolam, fentanyl, propofol, succinylcholine, esmolol, and sevoflurane) and intubation were uneventful.

Prior to prepping and during manipulation of her knees, the patient had sudden-onset hypoxia and hypotension, and her vital signs were HR 145 from 105, BP 86/46 from 149/131, and O2 sat 71% from 100% on ventilator FiO2 96%, and her EtCO2 decreased to 12 from 39. The patient was manually ventilated and noted to have good tidal volumes, reasonable compliance, and clear, bilateral breath sounds. Assistance was requested to assist with temporizing and diagnosing the underlying condition. Albuterol was given, and a fiberoptic bronchoscope demonstrated clear airways with endotracheal tube above the carina. A pneumothorax was ruled out with CXR given the recent interscalene block and history of trauma. The vital signs did not improve. 200 mcg epinephrine was administered, arterial and central lines were placed, and an intraoperative TEE was performed emergently. Her vital signs immediately improved: HR 121, BP 141/85, O2 sat 100% on ventilator FiO2 96%, EtCO2 27.

TEE showed dilated right ventricle (RV), severe RV dysfunction that spared the RV apex (McConnell's sign), a large patent foramen ovale (PFO) with right-to-left shunting, and a large PE with subtotal occlusion of the right pulmonary artery (PA); the left ventricle (LV) was normal. The PE Response Team, Cardiothoracic surgery (CS), Interventional Radiology (IR), and CSICU were consulted. The surgery was aborted, and the patient was transferred to the CSICU intubated. She was stabilized and then transferred to the IR suite for catheter-directed injection of tissue plasminogen activator and thrombectomy. A large occlusive embolus was found in the main right lower lobe PA, and the procedure improved PA pressures (54/24 to 48/20) and flow into the subsegmental branches of the right lower lung. No inotropic support was needed. The patient was left intubated and transferred back to CSICU on a heparin infusion. Her hospital course was complicated by thrombocytopenia and she was started on argatroban infusion due to concern for heparin induced thrombocytopenia. An inferior vena cava filter was placed, and several days later, the patient was transferred to the floor and underwent the ORIFs without any complications. Duplex ultrasound did not reveal any residual deep vein thrombosis (DVT). Transcranial Doppler ultrasound during hospital course revealed shunting, high intensity transient signals, and possible emboli. Plans were made for percutaneous closure of the PFO a week later.

## 3. Discussion

The incidence of PE is 0.7% to 30% after orthopedic surgery, and 2.3% to 6.2% after trauma [5]. Although our patient did not have a prior history of DVT/PE, she had several risk factors including obesity (BMI 50), smoking, limited mobility/venous stasis due to body habitus, and a recent knee and ankle injury. Undergoing a subsequent major trauma, acute inflammation, activation of the clotting cascade, and mobilization of both lower extremities in the operating room (OR) likely precipitated the PE. Intraoperatively, our patient presented with acute hemodynamic changes: tachycardia, hypotension, hypoxemia, and reduction of EtCO2 prior to prepping. If patients are hemodynamically stable and not undergoing surgery, the preferred confirmatory diagnostic tests for PE are ventilation/perfusion scan, angiography, and/or spiral computed tomography (CT) scan [1–4]. TEE is not generally used first line to diagnose PE. However, our patient developed unexplained and persistent hemodynamic disturbances, refractory to medical management. Thus, rescue TEE was performed to see if ventricular dysfunction was the culprit. Of note, the pneumothorax was excluded with CXR.

Indications for intraoperative use of TEE during noncardiac surgery have been stratified into categories based on level/strength of scientific evidence by the American Society of Echocardiography and Society of Cardiovascular Anesthesiologists. Category I indications (or indications with the highest level of evidence) include using rescue TEE for diagnostic evaluation of acute, persistent, and life-threatening hemodynamic disturbances unresponsive to treatment. Rescue TEE is emergent and unanticipated and used in “rescue situations” to guide medical management or surgical intervention. In our patient, TEE was used to assess her ventricular function in real-time, visualize possible new global or regional LV or RV dysfunction, and rule out other life-threatening diagnoses like pericardial tamponade or hemoperitoneum from trauma [6–8].

**Figure 1:**
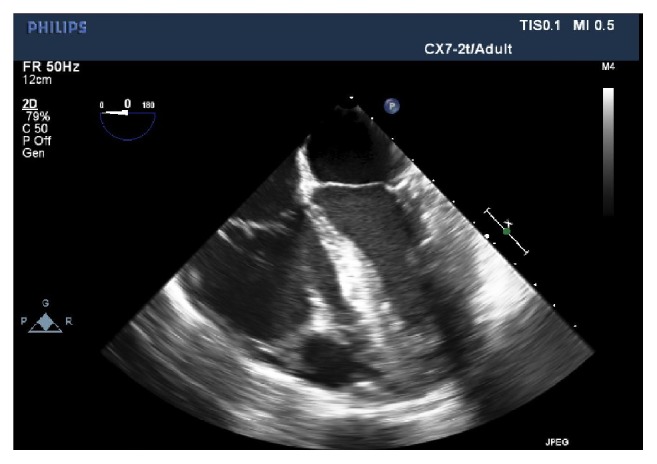
TEE mid-esophageal 4 chamber view showing dilated RV, RV end-diastolic diameter 48 mm.

**Figure 2:**
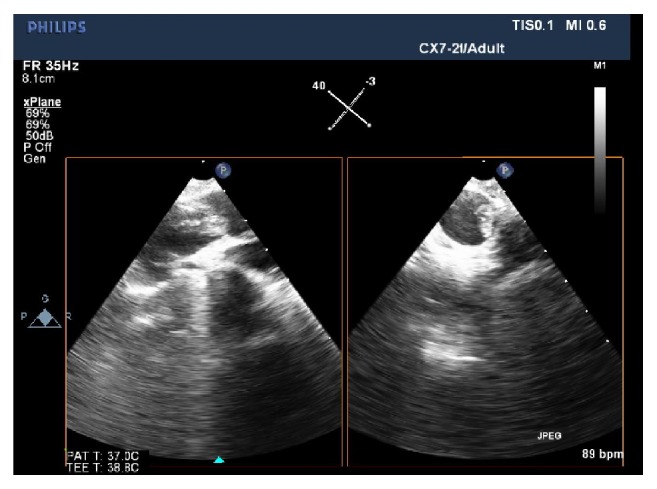
TEE mid-esophageal view of PA with x-plane mode showing large embolus with subtotal occlusion in right PA.

Our patient's TEE showed a dilated RV with a RV end-diastolic diameter (EDD) 48 mm (Figure 1). The most common finding on TEE in patients with PE is RV dilation, defined as RV EDD >27 mm or RV/LV EDD ratio >0.7, with ratio >1 associated with higher morbidity. Other common echo findings associated with PE include bowing of the interventricular septum, tricuspid regurgitation, and McConnell's sign which was seen in our patient, suggesting acute right ventricular pressure overload and an underfilled LV [9–12]. A large PE with subtotal occlusion in the right PA was also seen in her TEE (Figure 2). TEE can also directly visualize emboli in the right heart or pulmonary vasculature [9], but limitations exist. Rosenberger et al.'s study performed intraoperative TEE exams and reviewed their findings on patients with known PE immediately before pulmonary embolectomy. TEE correctly demonstrated the presence of PE in 46% of patients. Emboli were visible on TEE in only 26% of patients, with the lowest sensitivity in the left PA. Vieillard-Baron et al. showed increasing sensitivity and specificity for detecting proximal PE, as emboli were rarely seen in distal pulmonary vasculature. However, signs of PA obstruction, RV dysfunction, leftward interatrial septal bowing, and tricuspid regurgitation, were seen in 96%, 98%, and 50% of TEE findings. The use of intraoperative TEE to visualize emboli in PE is limited, but the use of TEE to detect signs of PA obstruction in PE can be beneficial [13, 14]. Furthermore, researchers found the diagnosis of PE with TEE to be fast and accurate, with an average of 9.6 minutes, sensitivity of 80.5%, and specificity of 97.2%, comparing favorably to spiral CT [15]. TEE is readily available and portable in the OR and can be performed without stopping surgery or resuscitation efforts.

In addition to PE, the most common intraoperative TEE findings in noncardiac surgery include hypovolemia, low EF, regional wall motion abnormality, and RV failure. Studies have shown intraoperative TEE to be beneficial for establishing a diagnosis or changing medical and surgical management in up to 80% of patients, including directing treatments for myocardial infarction (MI), valve pathology, and ventricular failure. In one study, presumptive diagnoses were made in 19 of 22 patients with TEE, including emboli, MI, and pericardial tamponade [16–19].

TEE has also been used occasionally in orthopedic surgeries with risk for fat or cement embolism including total hip arthroplasty (THA). For example, TEE may detect PE from fat or bone debris during intramedullary reaming or depression in ventricular function secondary to bone cement. It may be beneficial to recommend prophylactic TEE for monitoring high-risk patients during noncardiac surgery, i.e., patients with multiple risk factors for PE and coexisting coronary artery disease, previous MI, or unstable angina with ventricular dysfunction undergoing THA. Currently, orthopedic surgery is a Category III indication [13, 20, 21].

**Figure 3:**
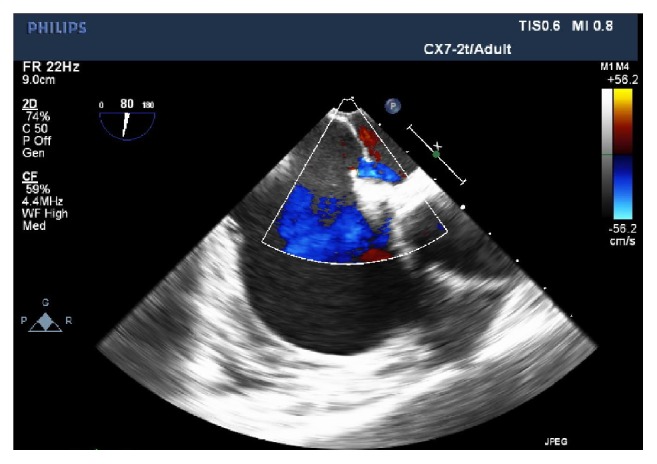
TEE mid-esophageal RV inflow-outflow view with color flow Doppler showing atria septum consistent with PFO (predominant R-to-L shunting).

Finally, TEE findings in our patient showed an incidental, previously undiagnosed PFO (Figure 3). Our presentation is similar to a case report of an 89-year-old male whose ORIF intertrochanteric fracture was aborted after he developed sudden O2 desaturation to 85% without a drop in EtCO2 during closed reduction and manipulation of the fracture (before incision). Intraoperative TEE was unavailable at the facility, and his PE was diagnosed by spiral CT immediately after stopping surgery. A previously undiagnosed PFO was also found. In both instances, a large PE would increase the afterload to the RV, increase right heart pressure, and increase the right-to-left shunting through the PFO, ultimately worsening the patient's hypoxemia. However, the increased flow to the left atrium through the shunt would maintain some left heart volume and systemic perfusion, partly offsetting the sudden-onset hemodynamic insult (possibly explaining why a drop in EtCO2 was not seen in the 89-year-old) [22].

A case of PE diagnosed intraoperatively by rescue TEE in a morbidly obese patient undergoing orthopedic surgery following motor vehicle crash is described. This patient developed unanticipated, acute, and life-threatening hemodynamic changes refractory to medical management. Using rescue TEE, RV dysfunction, and a large PE was visualized which led to our patient receiving catheter-directed tPA injection and thrombectomy. Intraoperative rescue TEE is essential when PE is suspected in the setting of hemodynamic instability. Nonetheless, more studies need to be completed to ultimately establish formal algorithms for the management of perioperative PE.
